# Case of Small-Pox after Small-Pox from Inoculation

**Published:** 1826-01

**Authors:** Thomas Richards


### Art. VI.—*Case of Small-Pox after Small-Pox from Inoculation.* By Thomas Richards, Esq.

As the following case may be deemed somewhat interesting,—especially as there have been recently some animated discussions on the subject to which it refers, in the Royal Academy of Medicine at Paris,—I am induced to place it before the public, through the medium of your Journal.

On Sunday, October 30th, my friend, Mr. Adams, of Charlotte-street, was called to see Mrs. M., aged twenty-two, and residing at No. 6, Pitt-street, Fitzroy-square. He found her labouring under a considerable degree of fever, with a pustular eruption on her face, head, arms, and other parts of her body, in every respect resembling that of small-pox. Upon inquiry, however, it appeared that, about fifteen years ago, she was inoculated at Milverton, in Somersetshire, with variolous matter; the cicatrix of which is still visible on the left arm. From this inoculation she had the small-pox; and a letter, subsequently received from her father, corroborates most circumstantially this statement, as he decidedly affirms that she has had, not only the small-pox, but the chicken-pox, and every other infectious disease usually incident to youth. From the patient's statement, and from the appearance of the disease, we were induced to pay a very close attention to the case; the particulars of which are now submitted to the profession.

The eruption made its appearance on Saturday, the day before Mr. Adams saw the patient. For some time previous to its appearance, Mrs. M. had been indisposed, and her indisposition increased till Friday the 28th of October, bearing all the symptoms of acute fever. On Friday, it had arrived at a sort of crisis; and on Saturday the eruption burst out, accompanied by a mitigation of the more violent febrile symptoms, although there was still a good deal of excitement remaining. On Sunday, the face, arms, head, chest, and other parts of the body, were covered with pustules; and the tongue and eyes were affected with the eruption. On Monday, some of the pustules had put on the appearance of maturation, while others retained their pristine character; and others, again, began to fade, without any purulent formation. On Tuesday, the same appearances presented themselves; the febrile symptoms having considerably abated, and the patient complaining of the lassitude which usually accompanies "the term" of the disorder. For the next four days, the pustules continued to die away; and, by the Monday following, (the ninth day from the appearance of the eruption,) the pustules had entirely disappeared, leaving only that discoloration of the skin which marked their situation. It is needless to add, that the patient did well, as she began to mend as soon as the eruption appeared, and continued to do so till it had completely vanished.

When we ascertained that Mrs. M. had had the small-pox before, our first opinion was, that her present malady might be an aggravated case of varicella; but the unequivocal character of the pustules, and the extreme degree of fever with which they had been ushered in, together with the present state of the patient's sufferings, induced us to abandon this, and to decide that it was a case of small-pox, modified and materially influenced by previous inoculation.* In this we were strengthened

---

* In a discussion on the subject of the varioloid epidemic now raging at Paris, M. Husson said, in the Royal Academy of Medicine in that city, that one day M. Leroy brought a list of twenty vaccinated persons, whom he declared to be labouring under small-pox. He (M. Husson) visited them all, and satisfied himself *that there was not a single case of true small-pox among them.*—Quære: Were not these cases of a modified and altered disease?

I remember a case after vaccination, similar to that which I have detailed, occurring about seven or eight years ago in the same neighbourhood; an account of which was, I believe, published in one of the Journals at the time.

by the concurring opinion of two other gentlemen of considerable experience in practice, who visited and examined the patient. To render this opinion still more valid, we discovered that a child *in the next house* was at that time recovering from the true and natural small-pox; which child I myself saw, a day or two after Mr. Adams had been called in to Mrs. M.

*Charlotte-street, Fitzroy-square; Nov. 20th,* 1825.

